# Bone Marrow Failure due to Aplastic Anemia, Associated With Serous Fat Atrophy, and Treated With Allogeneic, Haploidentical Stem Cell Transplantation: A Case Report

**DOI:** 10.1155/crh/6526961

**Published:** 2024-11-16

**Authors:** Matthew J. Pisarcik, Cameron J. Oswalt, Eric D. Carlsen, Mitchell E. Horwitz

**Affiliations:** ^1^Division of Medical Oncology, Department of Medicine, Duke University School of Medicine, Durham, North Carolina, USA; ^2^Duke Cancer Institute, Durham, North Carolina, USA; ^3^Department of Pathology, Duke University School of Medicine, Durham, North Carolina, USA; ^4^Division of Hematologic Malignancies and Cellular Therapy, Department of Medicine, Duke University School of Medicine, Durham, North Carolina, USA

**Keywords:** aplastic anemia, case report, gelatinous transformation of the bone marrow, haploidentical stem cell transplantation, levamisole, serous fat atrophy

## Abstract

We describe the case of a 27-year-old male, previously healthy though with a social history notable for recreational cocaine use, who developed bone marrow failure due to aplastic anemia (AA) with associated serous fat atrophy (SFA). After the SFA was corrected with nutritional supplementation, the patient underwent successful allogeneic, haploidentical stem cell transplantation with a regimen designed to treat AA. To our knowledge, this is the first case of hematopoietic stem cell transplantation (HSCT) performed following correction of SFA. Herein we propose our novel hypothesis that SFA, once resolved, is not a contraindication to stem cell transplantation, which we believe adds valuable insight toward an improved understanding of nutrition's role in HSCT. Additionally, the AA is thought to be toxin-induced and specifically levamisole-mediated after exposure to levamisole-adulterated cocaine. We highlight potential connections between levamisole, AA, and SFA and call for further efforts to understand these relationships—especially as the use of levamisole as a cocaine adulterant continues to rise across the globe.

## 1. Introduction

Hematopoietic stem cell transplantation (HSCT) is an effective treatment for bone marrow failure caused by aplastic anemia (AA), especially in younger individuals [1]. While HLA-matched donors are preferred, recent investigation showed cyclophosphamide-based post-transplantation graft-versus-host-disease (GVHD) prophylaxis conveys a favorable safety profile in the transplantation of cells from haploidentical donors [2]. This is particularly important for individuals from populations historically underrepresented in bone marrow transplantation as it indicates a key step in improving accessibility to HSCT for all patients.

Beyond AA, bone marrow failure has also been observed in serous fat atrophy (SFA), or gelatinous transformation of the bone marrow. SFA is a rare condition histologically characterized by degeneration of bone marrow adipocytes, hematopoietic stem cell loss, and stromal changes including deposition of hyaluronic acid–rich mucopolysaccharides [3]. While the exact pathophysiology is under investigation, SFA typically occurs in states of extreme catabolism or weight loss such as anorexia nervosa, malignancy, or infection [4]. Although uncommon, there is growing speculation SFA has been historically underrecognized; however, identification of SFA is paramount, as it can be reversed with nutritional supplementation and bone marrow stimulating agents [3, 5, 6]. Concurrence of SFA and AA is rare, though has been identified in both adult and pediatric populations, and there are no documented cases of HSCT (for treatment of AA or other processes) performed following biopsy-proven SFA [4, 7].

Herein, we present a case of bone marrow failure due to AA, associated with SFA, and ultimately managed with allogeneic, haploidentical HSCT. To our knowledge, this represents the first HSCT performed after SFA. Additionally, levamisole was detected in the AA workup, leading to our hypothesis that the patient's AA was toxin-mediated related to levamisole exposure. We believe this case highlights the need for improved understanding of the relationships between levamisole, AA, and SFA.

## 2. Case Presentation

**Figure 1:**
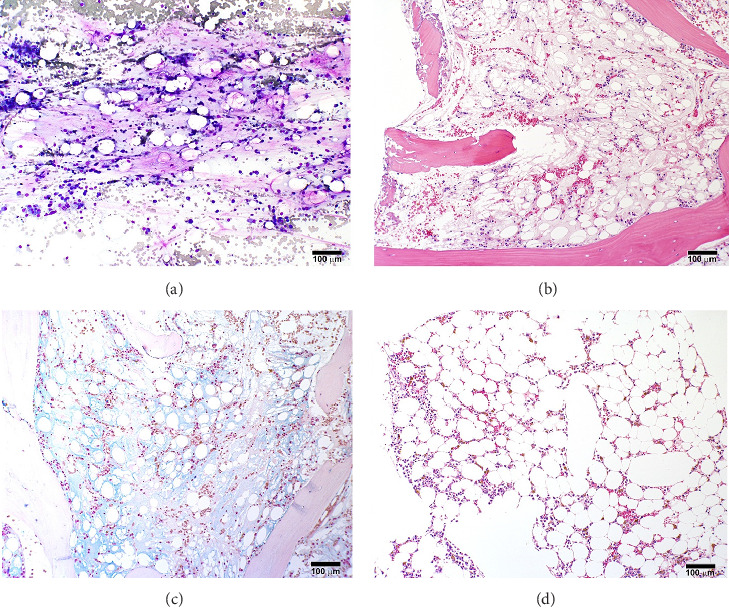
Bone marrow biopsy findings, day 8 and day 85. (a)–(c) represent findings from day 8; (d) represents findings from day 85. (a) Wright-stained bone marrow aspirate smear from hospital day 8, demonstrating markedly reduced hematopoietic elements, scattered necrotic adipocytes, and increased metachromatic seromucinous stromal material. (b) Hematoxylin and eosin (H&E)–stained section of the marrow core biopsy from hospital day 8, showing widespread adipocyte necrosis and gelatinous stromal changes. (c) An Alcian blue stain of the day 8 core biopsy shows an increase in hyaluronic acid–rich material. Alcian blue + hyaluronidase control was appropriate (not shown). (d) H&E-stained section from repeat bone marrow biopsy performed on hospital day 85, showing resolution of serous fat atrophy and improvement in myelopoiesis. However, maturing erythroid precursors and megakaryocytes are still very scarce.

A previously healthy 27-year-old male was transferred from an outside hospital for pancytopenia and several weeks of fever, mucosal bleeding, hematochezia, and painful lymphadenopathy, followed by a 20–30-pound weight loss. Family history was negative for telomere biology disorders. Social history was notable for cocaine use, and a qualitative serum assessment for levamisole was reported to be positive (attempts to obtain this laboratory result from the referring center outside of the United States were unsuccessful). Bone marrow biopsy revealed hypocellular marrow, extensive stromal injury, and early focal stromal changes worrisome for evolving SFA. The patient was pancytopenic on arrival to our institution and a repeat bone marrow biopsy demonstrated marked hypocellularity and extensive SFA (Figures 1(a), 1(b), and 1(c)). There was no evidence of hematolymphoid neoplasm, reticular fibrosis, or amyloid deposition. Screening for paroxysmal nocturnal hemoglobinuria and Fanconi anemia was negative, and karyotype and fluorescent in situ hybridization (FISH) analyses for chromosomal abnormalities associated with myeloid neoplasms were normal. A rheumatology consultation and workup were ordered; the evaluation was negative and there was low suspicion for an autoimmune-mediated process. Bone marrow stimulating agents and total parenteral nutrition (TPN) were initiated. The marrow stimulating agents included filgrastim-sndz; epoetin alfa; and romiplostim, followed later by eltrombopag. Due to persistent pancytopenia, a bone marrow biopsy was repeated one month later and demonstrated near complete resolution of SFA but persistent hypocellularity (5%). TPN was continued, though administration was complicated by concurrent carbapenem-resistant *Enterobacter cloacae* and *Staphylococcus epidermidis* bacteremia, prompting treatment with ceftazidime-avibactam and vancomycin.

A fourth bone marrow biopsy revealed patchy cellular marrow with improved cellularity (10%–30%), myeloid-predominant hematopoiesis, low-level erythroid maturation, and nearly undetectable megakaryocytes (Figure 1(d)). TPN was discontinued after roughly two months. However, the patient remained dependent on red blood cell and platelet transfusions. The patient was ultimately discharged with ongoing transfusion dependence; while hospitalized, he received a total of three months of filgrastim-sndz, four months of epoetin alfa, two months of romiplostim, and two months of eltrombopag. Several months later, the patient elected to pursue HSCT. The patient's brother was a haploidentical human leukocyte antigen match and served as the donor. A pretransplant conditioning regimen of rabbit antithymocyte globulin (brand name: Thymoglobulin), fludarabine, cyclophosphamide, and total body irradiation (400 cGy) was administered prior to delivery of 2.11 × 10^6^ bone marrow derived CD34+ cells/kg^2^. GVHD prophylaxis included post-transplant cyclophosphamide, mycophenolate, and tacrolimus. Day 31 engraftment studies demonstrated > 98% donor cells in both T-cell and myeloid lineages, and neutrophils and platelets engrafted within 30 days of transplantation. His transfusion requirements resolved three weeks post-transplant. As of twelve months post-transplant, he has been tapered off tacrolimus and has not required rehospitalization nor displayed signs of chronic GVHD.

## 3. Discussion

This case includes several notable features. To our knowledge, it is the first case of HSCT performed following correction of biopsy-proven SFA, prompting our novel hypothesis that SFA, once treated, is not a contraindication to HSCT. The case also highlights the successful utilization of a recently published HSCT preconditioning regimen intended for use in patients with acquired AA receiving cells from haploidentical donors, supporting the belief that this regimen can aid in efforts to further expand equitable HSCT access to all patients. Finally, we believe the AA was toxin-mediated, possibly secondary to levamisole exposure in the setting of adulterated cocaine use. Given the escalating use of levamisole as a cocaine adulterant on a global scale, we explore potential connections between levamisole, AA, and SFA and believe further efforts to understand these relationships are warranted [8].

Our patient's serial bone marrow biopsies provide intriguing insight into the dynamic nature of his bone marrow failure. The initial biopsy revealed marked hypocellularity with severe stromal injury and only early changes worrisome for SFA. Given the negative autoimmune and genetic evaluation, we hypothesize the bone marrow failure at this time was multifactorial due to a combination of toxin-mediated marrow injury and SFA. More specifically, the AA and SFA may have developed sequentially, with the AA preceding the SFA. This hypothesis stems from the patient's symptoms of pancytopenia preceding the weight loss by several weeks, with weight loss serving as the most likely trigger for SFA development [9]. However, this chronology is difficult to definitively confirm. Ensuing biopsies subsequently captured the progression and resolution of the SFA following administration of nutritional supplementation and marrow stimulating agents, consistent with prior approaches [3, 5]. However, despite the SFA's improvement, the marrow failure persisted. This further supports our hypothesis of a multifactorial bone marrow failure—specifically one not attributable to SFA alone.

Given persistent transfusion requirements despite SFA resolution and correction of malnutrition, the patient underwent allogeneic, haploidentical HSCT using an approach adapted for acquired AA [2]. There was considerable concern surrounding the patient's bone marrow integrity and ability to support robust hematopoiesis given the SFA resolved only a few months prior. However, the patient showed prompt hematopoietic recovery after transplant, including complete liberation from transfusion dependence, and his HSCT course was without major complication. To our knowledge, this represents the first case of HSCT performed in close temporal proximity to known SFA, driving our novel hypothesis that corrected SFA is not a contraindication to HSCT. This is particularly notable in the context of a similar, recently published case of a patient with AA secondary to severe, anorexia nervosa–mediated malnutrition who successfully underwent HSCT [10]. While both cases underscore the importance of nutritional support in the peri-HSCT period, there are differences between them. Specifically, despite the patient in the case described by Vanceviča and Žučenka having a body mass index (BMI) of 11 kg/m^2^ at the time of transplant, SFA was not appreciated on the preceding bone marrow biopsy [10]. Furthermore, Vanceviča and Žučenka highlight their successful HSCT course despite this low BMI at the time of transplant; in contrast, our patient's BMI improved from a low of 16 kg/m^2^ to 21 kg/m^2^ at the time of transplant. These cases together lend valuable insight toward the role of nutritional status in HSCT, especially given the barrier malnutrition has historically posed to HSCT.

As above, we believe the AA is most likely attributable to a toxic insult. This suspicion is driven by the detection of levamisole in the patient's serum, along with the lack of other clear AA risk factors including autoimmune phenomena or genetic abnormalities. Although levamisole is banned from use in humans in many countries due to its association with agranulocytosis, it remains prevalent globally due to its use as a cocaine adulterant, which is the most likely source of this patient's exposure [8, 11, 12]. Indeed, levamisole-adulterated cocaine has been increasingly associated with agranulocytosis as well as thrombocytopenia, despite thrombocytopenia not being an expected toxicity of cocaine itself [12, 13]. Although there are no previously documented cases of levamisole-induced AA, levamisole's associations with these other cytopenias drive our theory that a toxin-mediated marrow injury contributed to our patient's AA [8, 11].

Interestingly, levamisole is being explored in some countries as treatment for immune-mediated (rather than toxin-mediated) AA [14]. The proposed mechanism of action is particularly relevant to our patient's case. Bone marrow biopsies from patients with immune-mediated AA demonstrate abnormally elevated bone marrow adipocyte counts, which are hypothesized to impair hematopoiesis via destabilization of bone marrow microenvironment signaling pathways [15–17]. In an in vitro analysis by Liu et al., levamisole suppressed adipogenesis in immune-mediated AA; hence, the authors proposed levamisole may have a role in treating immune-mediated AA via adipogenesis suppression, recovery of the innate bone marrow microenvironment, and restoration of hematopoiesis [14, 17]. Although our patient's weight loss and malnutrition likely drove his SFA, levamisole-mediated adipogenesis suppression may have also contributed, culminating in SFA that was potentially multifactorial in nature. Of note, there are no previously documented cases of levamisole-associated SFA, perhaps due to the rarity of SFA or due to underreporting from levamisole being banned in many nations.

In conclusion, physicians should consider SFA as a cause of bone marrow failure in the setting of severe weight loss and promptly initiate nutritional supplementation and bone marrow stimulating agents if identified. Providers across disciplines should remain vigilant for adulterant-mediated complications in patients using illicit drugs, especially levamisole-mediated marrow injury in patients using cocaine with new cytopenias. Finally, this case suggests SFA, once treated, is not a contraindication to HSCT including from haploidentical donors. Further studies are required to confirm this hypothesis, and additional investigation into the relationships between levamisole, AA, and SFA is warranted.

## Data Availability

The data that support the findings of this study are available from the corresponding author upon reasonable request.
